# Sexual Dimorphism in Mandibular Ramus Morphometry: A Population-Specific Analysis Using Orthopantomograms in the Lucknow Region

**DOI:** 10.7759/cureus.80515

**Published:** 2025-03-13

**Authors:** Raghvendra Singh, Heena Singh, Nikhil Aggarwal, Garima Sehgal, Siddhartha Chandel, Navneet Kumar

**Affiliations:** 1 Forensic Medicine & Toxicology, Era's Lucknow Medical College and Hospital, Lucknow, IND; 2 Anatomy, King George's Medical University, Lucknow, IND; 3 Anatomy, Army College of Medical Sciences, New Delhi, IND; 4 Dentistry, Era's Lucknow Medical College and Hospital, Lucknow, IND

**Keywords:** anthropology, forensics, morphometry, radiography, skull, tomography

## Abstract

Introduction

Identification is a fundamental aspect of forensic anthropology, which is crucial for legal investigations, archaeological research, and disaster victim identification. The skull and pelvis exhibit significant sexual dimorphism to aid sex determination. In cases where the intact skull is unavailable, the mandible, particularly the ramus, serves as a vital bone for sex determination due to its distinct morphological characteristics.

Aim

To determine sex from the mandibular ramus using orthopantomograms (OPGs) and to develop a population-specific discriminant function for sex determination in the Lucknow region.

Materials and methods

This cross-sectional study was conducted from October 2024 to November 2024 and involved OPGs of 100 adults (55 males and 45 females) aged 20-50 years, selected randomly from patients referred to the Department of Dental Sciences at Era’s Medical College and Hospital, Lucknow. Measurements were done for the bigonial distance, projective ramus height, maximum ramus height, maximum anteroposterior diameter, and minimum anteroposterior diameter. Statistical analysis of data was done using IBM SPSS Statistics version 26.0 (IBM Corp., Armonk, NY), employing descriptive statistics, independent t-tests, and discriminant functions to evaluate the significance and accuracy of the measurements in sex determination.

Results

Significant sexual dimorphism was observed in various mandibular measurements, particularly bigonial distance, projective heights of the right and left ramus, and maximum heights of the right and left ramus (p < 0.001). The discriminant function achieved an overall accuracy of 96% in sex determination, with an accuracy of 94.5% in males and 97.8% in females.

Conclusion

The mandibular ramus is an effective tool for sex determination, exhibiting significant sexual dimorphism. Discriminant function has been developed to provide a reliable method of sex determination with high accuracy. Bigonial distance and projective ramus height were identified as the most significant parameters for sex determination. These findings contribute to forensic anthropology by offering a population-specific method for sex determination using mandibular ramus measurements and further support forensic investigations.

## Introduction

Identification facilitates the differentiation of individuals in various contexts, such as legal investigations, archaeological research, and victim identification in disasters. Among the different skeletal parts, the skull and pelvis are known to exhibit significant sexual dimorphism, which aids in sex determination. The skull, in particular, is considered the most dimorphic and easily identifiable portion of the skeleton for sex determination, offering up to 92% accuracy in identification [1,2]. However, in cases where the intact skull is not available, the mandible becomes a vital bone for sex determination due to its distinct morphological characteristics. As the largest and strongest facial bone, the mandible exhibits notable sexual dimorphism, particularly in its ramus. The mandibular ramus displays various measurable parameters, such as ramus height, bigonial width, and anteroposterior diameter, which differ significantly between males and females. Computed tomographic evaluations and digital radiographic studies have reinforced the mandibular ramus as a crucial tool for sex determination, with studies like those by Revanth et al. (2022) reporting significant differences in ramus measurements between sexes [3]. Additionally, population-specific analyses in Sriganganagar highlighted that maximum and projected ramus heights consistently provided reliable differentiation [4]. These differences in size and shape of the mandibular ramus form the basis for forensic identification. Recent studies have demonstrated that various parts of the mandible, such as the coronoid process and ramus, exhibit significant sexual dimorphism, making them highly reliable for forensic identification [5]. For instance, Alias et al. (2018) conducted morphometric analyses of CT-scanned mandibles, achieving high accuracy in sex determination [6].

Orthopantomograms (OPGs) or panoramic radiographs have emerged as useful tools for measuring the mandible's dimensions. OPGs offer a comprehensive view of the entire mandible and its anatomical landmarks, enabling precise measurement of various parameters. Furthermore, panoramic radiographic assessments of mandibular measurements, as demonstrated by Mostafa and El-Fotouh (2020), have shown significant correlations with sex differences, bolstering their application in forensic science [7]. Also, researchers have devised discriminant functions using parameters from different parts of the mandible to enhance the accuracy of sex determination [8]. Population-specific functions have proven particularly effective, as highlighted by Shakya et al. (2022) in Nepal, who reported 84% accuracy in sex determination using digital OPG measurements of the mandibular ramus [9]. Similarly, Esfehani et al. (2023) achieved high sensitivity and specificity in sex determination using mandibular indices in an Iranian population [10].

Several previous studies have highlighted the significance of the mandibular ramus in sex determination. For instance, More et al. (2017) conducted a morphometric analysis of the mandibular ramus in the Vadodara population and found significant differences in ramus dimensions between males and females, with an overall accuracy of 69% for diagnosing sex [5]. Similarly, Indira et al. (2012) conducted a study in the Bangalore population and reported significant sexual dimorphism in the mandibular ramus, achieving an overall accuracy of 76% for sex determination [11]. These studies underscore the potential of the mandibular ramus as a reliable indicator of sex, although the accuracy and significant parameters vary across populations.

The present study evaluated the usefulness of the mandibular ramus in sex determination using OPGs in the Lucknow population. The primary objectives were to measure and evaluate the dimensions of the mandibular ramus as observed on OPGs and to assess its efficacy as an aid in sex determination. The study contributes to the field of forensic anthropology by providing a population-specific method for sex determination using the mandibular ramus. The findings have the potential to enhance forensic investigations and support identification processes in various legal and archaeological contexts.

## Materials and methods

This cross-sectional observational study was conducted from October 2024 to November 2024 in the Department of Forensic Medicine and Toxicology in collaboration with the Department of Dental Sciences at Era’s Medical College and Hospital, Lucknow. The study group comprised OPGs of 100 randomly selected adults (55 males and 45 females) aged between 20 and 50 years who had reported for dental treatments. The sample size was determined based on a sample size calculation to ensure adequate statistical power. Using G*Power software version 3.1.9.7 (Heinrich Heine University Düsseldorf, Düsseldorf, Germany), a priori power analysis was conducted with an effect size of 0.5 (medium effect), alpha level of 0.05, and power (1-beta) of 0.80. The calculation indicated that a minimum sample size of 64 participants (32 males and 32 females) would be required to detect significant differences in mandibular measurements. To account for potential variability and ensure robust results, the sample size was increased to 100 participants. This approach ensured adequate representation of the population and meaningful statistical analysis, as supported by the high accuracy (96%) achieved in sex determination. Previous studies evaluating sexual dimorphism using mandibular parameters have employed similar sample sizes, making it a reliable benchmark for this research. Furthermore, the sample size was deemed sufficient to detect statistically significant differences in mandibular measurements between males and females, ensuring the robustness of the study's findings.

Informed consent was obtained from all participants, and ethical clearance was secured from the Institutional Ethics Committee, Era's Lucknow Medical College & Hospital, Era University, Lucknow (Reg. No.: ECR/717/Inst./UP/2015/RR-21; Approval No.: ELMC&H/R-CELL/2024/400).

The inclusion criteria included patients aged 20-50 years who were advised OPGs for dental treatments. Patients with developmental abnormalities, systemic conditions like hyperparathyroidism, or edentulous status were excluded. Additionally, images showing excessive loss of mandibular posterior teeth, over-erupted or tilted molars, trauma, surgically treated maxillofacial regions, artifacts, or technical faults were excluded to ensure measurement accuracy and avoid confounding factors.

**Figure 1:**
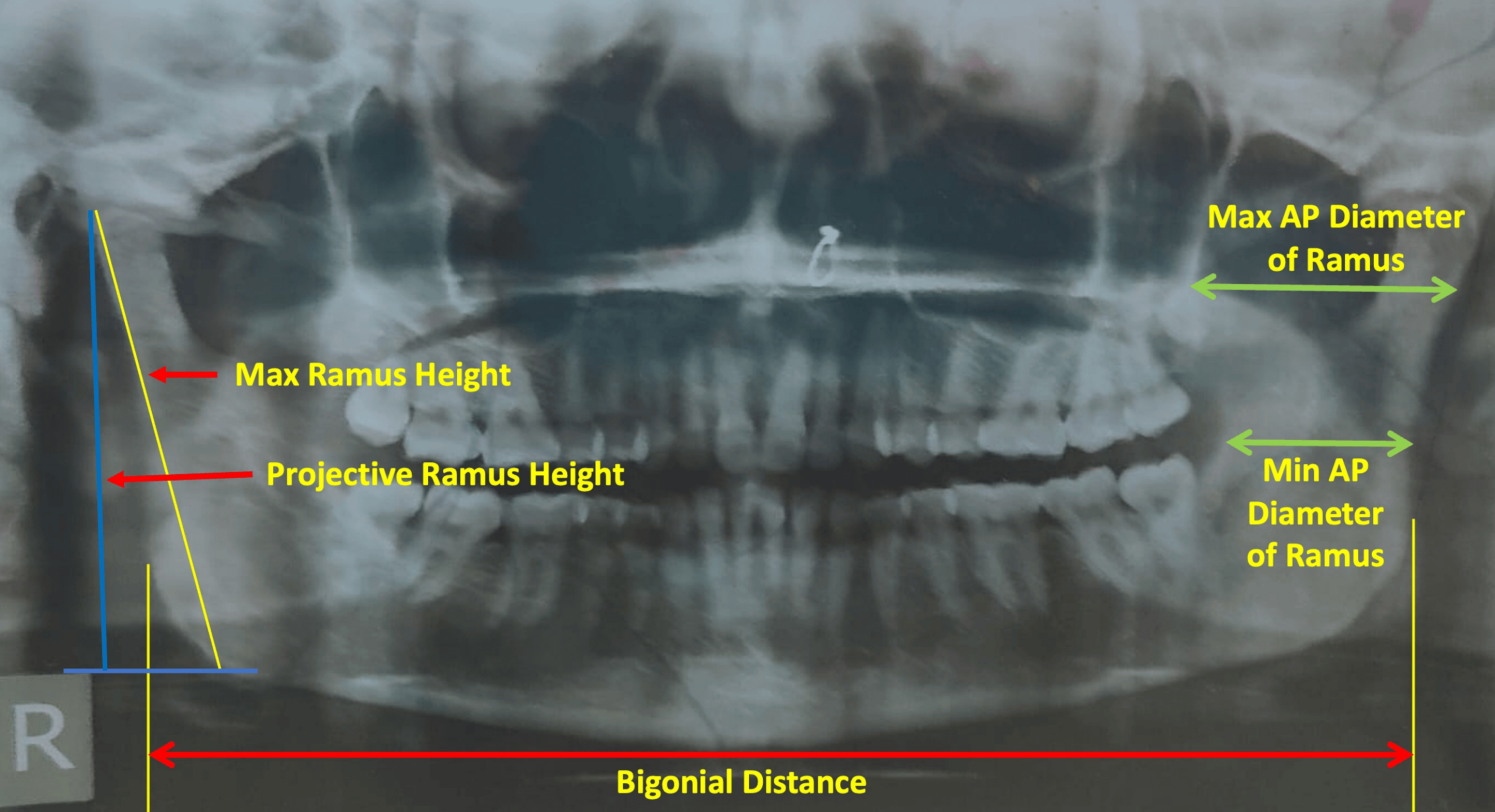
Orthopantomogram of the mandible showing labeled measurements (in cm), including bigonial distance, projective ramus height, maximum ramus height, and maximum and minimum anteroposterior (AP) diameter of the ramus.

Measurements were taken from the OPGs using digital vernier calipers to assess specific mandibular parameters. These included bigonial distance, which measures the distance between the two gonial angles of the mandible; projective ramus height, defined as the height from the superior-most point of the condyle to the horizontal line on the tubercle; maximum ramus height, which is the height from the condyle to the most inferior border of the ramus; and maximum and minimum anteroposterior diameters, representing the maximum and minimum widths of the ramus, respectively (Figure 1). All measurements were recorded in centimeters and analyzed to assess their role in determining sexual dimorphism. To ensure the accuracy and reliability of the measurements, all assessments were carried out by a single trained observer to minimize inter-observer bias. Additionally, measurements for 20 randomly selected OPGs were repeated at two different time intervals by the same observer to assess intra-observer consistency. The intra-class correlation coefficient (ICC) was calculated to evaluate measurement reliability, yielding an ICC value of 0.98 (95% CI: 0.96-0.99), indicating excellent agreement and minimal intra-observer variation. This high level of consistency confirms the accuracy and precision of the measurements, strengthening the robustness of the discriminant function analysis (DFA) results.

Statistical analysis

Categorical variables were summarized as N (%) and continuous variables as mean (SD). Comparisons of quantitative variables between two groups were performed using the Mann-Whitney U test or the unpaired t-test, as appropriate. For comparisons among three or more groups, the ANOVA test was applied. Box’s M test was used for sex verification, classification of function coefficients, and prediction accuracy. Statistical significance was determined at an alpha level of 0.05, with p-values <0.05 considered statistically significant. Data entry was conducted in a Microsoft Excel spreadsheet (Microsoft Corporation, Redmond, WA), and all analyses were performed using SPSS version 26 (IBM Corp., Armonk, NY).

## Results

The present study aimed to identify specific dimensions of the mandibular ramus exhibiting significant sexual dimorphism and to develop a population-specific discriminant function. Utilizing a cross-sectional design, OPGs of 100 adults from the Lucknow region were analyzed. The findings provide insights into sexual dimorphism in mandibular measurements and offer a reliable method for sex determination in this population. The study encompassed a total of 100 participants, consisting of 55 males (55.0%) and 45 females (45.0%). Males exhibited consistently higher measurements across all mandibular metrics compared to females, including bigonial distance, projective and maximum heights of both the right and left rami, as well as the maximum and minimum anteroposterior diameters. In contrast, females displayed relatively lower values across these parameters, highlighting significant sexual dimorphism in mandibular dimensions.

Significant differences between males and females in various mandibular measurements were observed. Bigonial distance, projective heights of the right and left ramus, as well as the maximum heights of the right and left ramus, demonstrated statistically significant variations between sexes. However, parameters such as the maximum and minimum anteroposterior (AP) diameters of the right and left ramus did not show statistically significant sex-based variations (p > 0.05) (Table 1).

**Table 1:** Test of equality of group means (Wilks' lambda) for significance. AP: anteroposterior.

Parameters (in cm)	Sex				p-value
	Male		Female		
	Mean	SD	Mean	SD	
Bigonial distance	18.16	0.57	16.48	0.61	<0.001
Projective height of the right ramus	7.09	0.51	5.64	0.38	<0.001
Projective height of the left ramus	7.06	0.55	5.73	0.43	<0.001
Maximum height of the right ramus	7.40	0.66	6.36	0.34	<0.001
Maximum height of the left ramus	7.47	0.75	6.42	0.39	<0.001
Maximum AP diameter of the right ramus	4.06	0.40	3.95	0.42	0.193
Maximum AP diameter of the left ramus	4.02	0.45	3.97	0.39	0.601
Minimum AP diameter of the right ramus	3.04	0.38	3.02	0.28	0.862
Minimum AP diameter of the left ramus	3.05	0.43	3.14	0.34	0.292

Using Box's M test, the assumption of equality of variance-covariance matrices was validated, confirming that the use of DFA was appropriate for distinguishing between male and female sexes based on mandibular ramus measurements. The analysis produced a statistically significant result (p < 0.001), highlighting distinct differences between male and female groups. These findings highlight the utility of mandibular ramus characteristics as effective indicators for sex differentiation.

The accuracy in discriminating sex was assessed using canonical discriminant function coefficients derived from mandibular ramus measurements (Table 2). These coefficients, along with the constant value, were used to establish a discriminant function for classifying individuals into male and female categories based on their mandibular dimensions. To ensure the model's robustness, a stepwise approach was considered to eliminate nondiscriminatory variables, but the initial analysis retained all variables to evaluate their individual contributions. Future studies may explore stepwise methods to optimize classification accuracy further.

**Table 2:** Canonical discriminant function coefficients for males and females. AP: anteroposterior.

Classification of function coefficients				
Parameters	Sex		Sectioning point	
	Male	Female	Male	Female
Bigonial distance	59.006	55.517	1.734	-2.120
Projective height of the right ramus	-1.339	-12.592		
Projective height of the left ramus	8.009	11.721		
Maximum height of the right ramus	-19.853	-14.870		
Maximum height of the left ramus	10.701	8.292		
Maximum AP diameter of the right ramus	40.933	38.906		
Maximum AP diameter of the left ramus	-25.471	-25.747		
Minimum AP diameter of the right ramus	48.914	49.287		
Minimum AP diameter of the left ramus	-22.591	-21.778		
(Constant)	-598.002	-501.677		
Fisher's linear discriminant functions				

The established sectioning points for sex classification were identified as 1.734 for males and -2.120 for females. In this context, when the discriminant function value approaches 1.734, the individual is more likely to be classified as male, while proximity to -2.120 suggests a higher probability of categorization as female (Table 2).

The sex classification accuracy based on mandibular measurements was high (Table 3). The original cases were used to test the classification accuracy of the discriminant function. For males (n = 55), the accuracy reached 94.5%, correctly identifying 52 individuals. Among females (n = 45), the accuracy was 97.8%, accurately classifying 44 individuals. Overall, for the total sample (n = 100), the accuracy stood at 96%, indicating the effectiveness of these measurements in distinguishing between sexes.

**Table 3:** Prediction accuracy.

Particulars	Predicted accuracy (%)
Male (n = 55)	52 (94.5%)
Female (n = 45)	44 (97.8%)
Total (n = 100)	96 (96%)

The coefficients reveal the varying degrees of influence exerted by specific mandibular measurements on the dependent variable (Table 4). Bigonial distance emerges as a key contributor, with an unstandardized coefficient of 0.905, underscoring its significant impact on the outcome. The projective heights of the right (2.920) and left (-0.963) ramus demonstrate notable coefficients, further emphasizing their influence. The maximum heights of the right (-1.293) and left (0.625) ramus display moderate coefficients, indicating a lesser but still meaningful effect. In contrast, the maximum and minimum anteroposterior diameters exhibit smaller coefficients, suggesting a relatively weaker impact within the model. Finally, the constant term, with a value of -25.185, represents the baseline outcome of the dependent variable when all mandibular measurements are zero.

**Table 4:** Descriptive analysis. AP: anteroposterior.

Variables	Unstandardized coefficients	Standardized coefficients	Structure coefficients
Bigonial distance	0.905	0.533	0.825
Projective height of the right ramus	2.920	1.333	0.737
Projective height of the left ramus	-0.963	-0.484	0.690
Maximum height of the right ramus	-1.293	-0.703	0.497
Maximum height of the left ramus	0.625	0.385	0.446
Maximum AP diameter of the right ramus	0.526	0.215	0.068
Maximum AP diameter of the left ramus	0.072	0.031	-0.055
Minimum AP diameter of the right ramus	-0.097	-0.033	0.027
Minimum AP diameter of the left ramus	-0.211	-0.082	0.009
(Constant)	-25.185	-	-

## Discussion

The study aimed to establish sex differences in the mandibular ramus using OPGs and to evaluate its usefulness as a tool for sex determination in the Lucknow population. The results demonstrate significant sexual dimorphism in various mandibular measurements, particularly in the bigonial distance, projective heights of the right and left ramus, and maximum heights of the right and left ramus. These findings align with previous studies that have highlighted the potential of mandibular ramus measurements in sex determination.

More et al. (2017) conducted a morphometric analysis of the mandibular ramus in the Vadodara population and found significant differences in ramus dimensions between males and females. Their study reported an overall accuracy of 69% for diagnosing sex, with the highest univariate sexual dimorphism observed in the maximum ramus breadth and projective ramus height [5]. In comparison, our study achieved a higher overall accuracy of 96%, with the bigonial distance and projective ramus height being the most significant parameters for sex determination in the Lucknow population. Indira et al. (2012) conducted a similar study in the Bangalore population and reported significant sexual dimorphism in the mandibular ramus, achieving an overall accuracy of 76% for sex determination [11]. Their study identified minimum ramus breadth, condylar height, and projective height of the ramus as the most significant parameters. Mandibular ramus indices, particularly coronoid and condylar heights, have repeatedly shown statistical significance in sex differentiation. Supriya et al. (2023) examined mandibular ramus measurements in the West Godavari population, reporting high reliability of condylar height and coronoid process as indicators for sex differentiation [12]. Their study confirms that panoramic radiography is an effective forensic tool. A study conducted by Singh et al. (2023) emphasized the utility of the mandibular ramus and gonial angle measurements for sex determination using digital panoramic radiography. They reported significant differences in projective ramus height, minimum ramus breadth, and coronoid ramus height, underscoring their role in forensic applications [13]. A study by Mostafa & El-Fotouh (2020) highlighted coronoid length and ramus breadth as reliable markers, particularly in Egyptian populations [7]. Our study corroborates the importance of projective height but highlights bigonial distance as a more significant parameter in the Lucknow population.

Arthanari et al. (2024) conducted a systematic review and meta-analysis, confirming that projective ramus height and other ramus parameters exhibit significant sexual dimorphism [14]. The study highlighted the need for population-specific standards to optimize forensic accuracy. Also, Alves et al. (2022) systematically reviewed metric analysis of the mandibular ramus and its angular parameters, showing strong dimorphism, particularly in bicondylar angle and coronoid process height, across various populations [15]. Furthermore, Saloni et al. (2020) conducted a study in the Sriganganagar population and reported that the mean values of minimum ramus breadth, maximum ramus height, and projected ramus height were significantly higher in males, with an overall accuracy of 77.6% for sex determination [4]. The present study has a higher accuracy, suggesting that the discriminant function developed for the Lucknow population may be more effective in this specific geographical context. Ranaweera et al. (2020) reported statistically significant relationships between mandibular ramus dimensions and sex, achieving a total classification accuracy of 75.4% using discriminant analysis, with condylar height being the most reliable measure [16]. Similarly, Koju et al. (2021) noted that projective ramus height demonstrated the highest dimorphism in their study on a Nepalese sample [17]. Premkumar et al. (2023) analyzed mandibular ramus flexure in a South Indian population and found moderate predictive accuracy (58.5%) for sex determination [18]. They recommend using this trait as a supplementary tool in conjunction with other indicators. Shakya et al. (2022) conducted a study in the Lalitpur population in Nepal and reported statistically significant higher measurements in males for all mandibular parameters, with condylar height being the most significant predictor for determining sex [9]. The discriminant function equation derived in their study achieved an overall accuracy of 84%. Our study, with an accuracy of 96%, highlights the importance of developing population-specific discriminant functions to achieve higher accuracy in sex determination.

Digital OPG and CT imaging have shown significant utility in sex determination. Revanth et al. (2022) demonstrated that mandibular ramus measurements using CT yielded a prediction accuracy of 96.4%, highlighting the value of condylar height and maximum ramus width [3]. The findings of the current study have significant implications for forensic anthropology and legal investigations in the Lucknow region. The high accuracy of 96% for sex determination based on mandibular measurements indicates the reliability of the mandibular ramus as an indicator of sex. The study identifies the bigonial distance and projective ramus height as the most significant parameters, which can be used to develop a discriminant function specific to the Lucknow population. The development of population-specific discriminant functions is crucial for forensic investigations, as the accuracy of sex determination can vary significantly across different populations. Ingaleshwar et al. (2023) compared panoramic radiographs across populations and demonstrated significant differences in maximum ramus breadth and coronoid height, suggesting OPG as an effective alternative to CT in routine forensic settings [19]. Also, Behl et al. (2023) conducted a radiographic analysis in North India, demonstrating the significant utility of condylar and coronoid heights for sex estimation with an accuracy exceeding 80%. This highlights the relevance of mandibular indices for forensic purposes in diverse populations [20]. The current study contributes to the existing body of knowledge by providing a reliable method for sex determination using mandibular ramus measurements in the Lucknow population. The findings can support forensic experts in identifying individuals in various contexts, such as disaster victim identification, legal investigations, and archaeological research.

Limitations

While this study achieved high accuracy in sex determination, it is essential to acknowledge its limitations. The study's sample size of 100 participants may not fully represent the entire Lucknow population. Future studies with larger and more diverse samples are recommended to validate the findings and further refine the discriminant function. Additionally, the study focused on a specific age range (20-50 years) to minimize the influence of age-related changes on mandibular measurements. However, future research could explore the applicability of the discriminant function across different age groups to enhance its utility in various forensic contexts. The study also highlighted the importance of considering environmental and social factors that may influence the development and structure of the mandible. Further research is needed to investigate the impact of these factors on mandibular measurements and their implications for sex determination.

## Conclusions

The mandibular ramus serves as an effective tool for sex determination, exhibiting significant sexual dimorphism in various measurements. This study identified the bigonial distance and projective ramus height as the most significant parameters for sex determination in the Lucknow population. The developed discriminant function achieved an overall accuracy of 96%, highlighting the reliability of mandibular ramus measurements in forensic investigations. The findings of this study contribute to the field of forensic anthropology by providing a population-specific method for sex determination using the mandibular ramus. The high accuracy achieved underscores the importance of developing discriminant functions tailored to specific populations to enhance the reliability of forensic identification. Future research with larger and more diverse samples is recommended to validate the findings and further refine the discriminant function.
